# Dynamic roles of inflammasomes in inflammatory tumor microenvironment

**DOI:** 10.1038/s41698-021-00154-7

**Published:** 2021-03-08

**Authors:** Jeong-Hoon Jang, Do-Hee Kim, Young-Joon Surh

**Affiliations:** 1 Tumor Microenvironment Global Core Research Center, College of Pharmacy, Seoul National University, Seoul, South Korea; 2 Department of Chemistry, College of Convergence and Integrated Science, Kyonggi University, Suwon, Gyeonggi-do South Korea; 3 Department of Molecular Medicine and Biopharmaceutical Sciences, Graduate School of Convergence Science and Technology, Seoul National University, Seoul, South Korea; 4 Cancer Research Institute, Seoul National University, Seoul, South Korea

**Keywords:** Cancer prevention, Cancer prevention

## Abstract

The inflammatory tumor microenvironment has been known to be closely connected to all stages of cancer development, including initiation, promotion, and progression. Systemic inflammation in the tumor microenvironment is increasingly being recognized as an important prognostic marker in cancer patients. Inflammasomes are master regulators in the first line of host defense for the initiation of innate immune responses. Inflammasomes sense pathogen-associated molecular patterns and damage-associated molecular patterns, following recruitment of immune cells into infection sites. Therefore, dysregulated expression/activation of inflammasomes is implicated in pathogenesis of diverse inflammatory disorders. Recent studies have demonstrated that inflammasomes play a vital role in regulating the development and progression of cancer. This review focuses on fate-determining roles of the inflammasomes and the principal downstream effector cytokine, IL-1β, in the tumor microenvironment.

## Introduction

The complexity of the tumor microenvironment is reflected by not only heterogeneity of cancer cells but variable composition of their surrounding stromal cells^1^. The profile of infiltrated/resident immune cells and inflammatory mediators in the proximity of cancer cells defines the inflammatory tumor microenvironment^2^. The inflammatory microenvironment is now recognized as an important participant or a regulator of all stages of tumor development, from an early stage (initiation) of carcinogenesis to tumor promotion and metastatic spread to distant organs^1–4^.

**Fig. 1:**
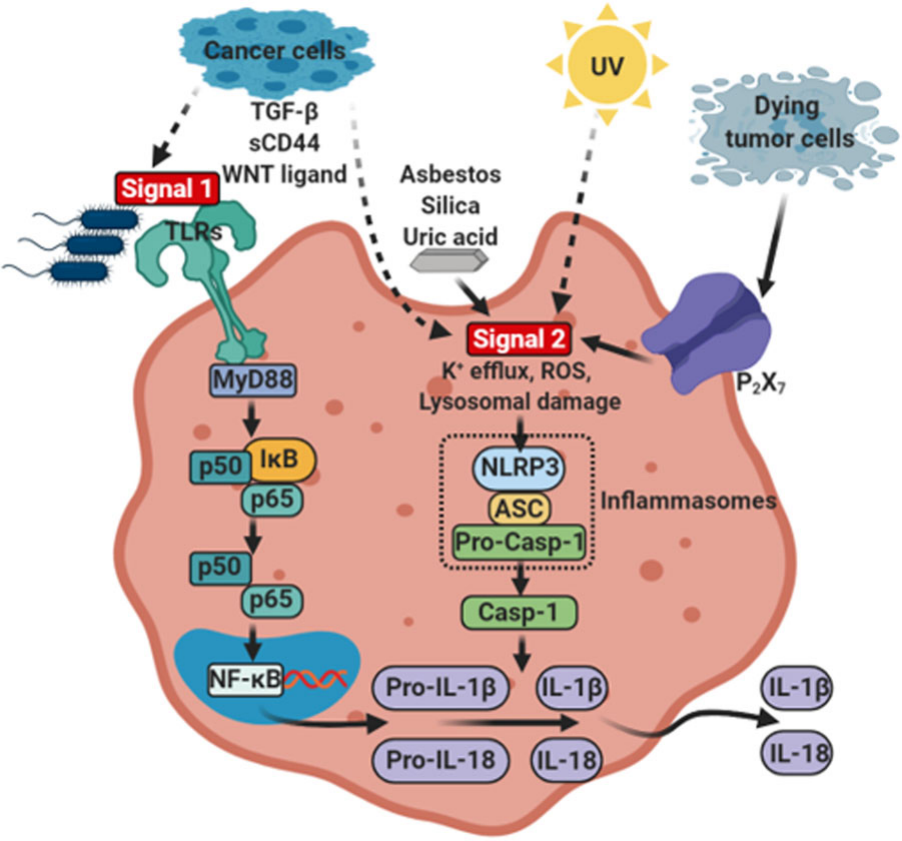
Mechanisms of conventional inflammasome activation and alternative inflammasome activation in tumor microenvironment. Conventional inflammasome activation process comprises two main signals: Signal 1, which is induced by PRRs such as Toll-like receptors activated by PAMPs such as bacterial lipopolysaccharide (LPS). This leads to the transcriptional upregulation of *IL1B*, and *IL18* via NF-κB signaling. Signal 2 is provided by PAMPs or DAMPs, such as ATP and crystals (e.g., asbestos, silica, or uric acid), which activates signaling events including K^+^ efflux, reactive oxygen species (ROS), and lysosomal damage, leading to activation and recruitment of NLRP3 oligomerization and formation of NLRP3 inflammasome complex. The activation and formation of NLRP3 inflammasome provoke auto-cleavage of the active caspase-1, which then proteolytically cleaves pro-IL-1β and pro-IL-18 into their bioactive forms IL-1β and IL-18. Alternatively, the inflammasomes can be activated by cancer cell-derived soluble factors (e.g., TGF-β, sCD44, and WNT ligand), ATP from dying tumor cells, and UV in tumor microenvironment. This alternative activation of inflammasome can also include maturation and secretion of IL-1β and IL-18 in tumor microenvironment.

The inflammasomes are multi-protein complexes, composed of an NOD-like receptor, apoptosis-associated speck-like protein containing a CARD domain (ASC), and pro-caspase-1, which are assembled upon pathogen- or damage-associated stimuli^5^. Activation of the inflammasomes accompanies the cleavage of pro-interleukin (IL)-1β and pro-IL-18 by caspase-1 into their active forms (Fig. 1). This triggers host-protective responses by promoting infiltration of inflammatory and immunocompetent cells into the inflamed site in the innate immunity^5^. Due to the critical roles of host immune defense against tissue damages or microbial infections, the inflammasomes are well-recognized as important and indispensable protein complexes for maintaining homeostasis.

Although the inflammasomes mainly exert their beneficial roles in the innate immune defense, aberrant activation of the inflammasomes and concurrent overexpression/overproduction of their effector molecules have been observed in several types of human malignancies^6^. The inflammasomes are expressed in various types of cells, such as T and B lymphocytes, antigen-presenting cells, and even cancer cells in the tumor microenvironment. The effects of the inflammasomes on cancer development are influenced by several factors^6,7^. In this review, we will discuss cell type- and microenvironmental condition-dependent roles of the inflammasomes and their principal effector molecule, IL-1β, in the development and progression of various types of cancers (Table 1).

**Table 1:** Effects of cell type-dependent expression of inflammasome components in tumor microenvironment.

Cancer type	Inflammasome components	Cell type	Roles	Ref.
Breast cancer	IL-1β	CD11c^+^DCs	Tumor progression	^30^
	IL-1β	Macrophages	Primary tumor growth & pulmonary metastasis	^18^
	NLRC4/IL-1β	F4/80^+^/CD11b^+^ macrophages	Tumor growth & induction of *Vegfa* expression in obesity-driven breast cancer mouse model	^35^
	IL-1β	Innate immune cells	Prevention of metastatic colonization of MICs	^36^
Colorectal cancer	Nlrp3	Hematopoietic-derived cells	Prevention of colonic lesion formation, colon inflammation, hyperplasia, and dysplasia	^43^
	Nlrp6	Hematopoietic-derived cells	Inhibition of tumor burden	^45^
	Nlrp6	Epithelial & stromal cells	No role in limiting tumor burden	^45^
	Nlrc4	Hematopoietic-derived cells	No effect on CAC	^46^
	Nlrc4	Epithelial & stromal cells	Prevention of tumor burden	^46^
	IL-1β	Neutrophils	Induction of mucosa damage & tumor formation	^48^
Lung cancer	IL-1β	Neutrophils	Reduced NF-kB inhibitor efficacy	^60^
	IL-1β	Lung cancer cells	Increased development of lung metastasis	^62^
Skin cancer	IL-1β	Melanoma	Promotion of tumor growth, invasiveness, and metastatic potential	^71,72^
	ASC	Myeloid cells	Induction of tumor formation	^74^
	ASC	Keratinocytes	Inhibition of tumor formation	^74^
Hepatocellular carcinoma	IL-1β	Macrophages	Induction of EMT and pulmonary metastasis	^80^

## Fundamental roles of the inflammasomes in the innate immunity

The immune system initiates clearance of pathogens and repairs damaged tissues upon recognition of microbial infection, injuries, etc., thereby restoring cellular homeostasis. One of the key complexes essential for the operation of such innate immune responses is the inflammasomes. The innate immune system employs an array of germline-encoded pattern-recognition receptors (PRRs) to sense invariant microbial motifs^8^. PRRs are expressed in many different types of cells which are at the front line of defense, including macrophages, monocytes, dendritic cells, neutrophils, and epithelial cells^8^. As important components of inflammasomes, the PRRs contain the nucleotide-binding domain, the leucine-rich repeat containing proteins (also known as NOD-like receptors, NLRs) and the absent in melanoma 2 (AIM)-like receptor (ALRs). Upon inflammasome activation by sensing pathogen-associated molecular patterns (PAMPs) or damage-associated molecular patterns (DAMPs), NLRs, AIM2, or pyrin recruits ASC to form a multimeric complex. Oligomerized ASC subsequently recruits pro-caspase-1 to this complex, which is converted into catalytically active caspase-1 by proximity-induced self-cleavage. The bioactive caspase-1 subunits p20 and p10 then proteolytically cleave pro-IL-1β and pro-IL-18 to generate mature form of bioactive IL-1β and IL-18, respectively (Fig. 1). Activation of the inflammasomes triggers an alarm to alert adjacent cells and tissues by releasing pro-inflammatory cytokines. This ends up with amplification of inflammatory cascade by promoting recruitment of effector cell populations required for the immune responses and tissue repair^9^.

Although acute inflammation is a physiological response to infections or tissue damage, it must be properly resolved. Unresolved inflammation can lead to a chronic inflammatory state, which is implicated in the pathogenesis of a wide variety of human disorders including metabolic syndromes, autoimmune disorders, and cancer^9^. For the systematic termination of acute inflammation, it has now become evident that coordinated resolution programs start shortly after inflammatory responses begin^10^. Indeed, inflammatory cells in an early phase of inflammation undergo a functional re-polarization to get properly involved in the onset and establishment of resolution. The fine-tuning of inflammatory switch allows an effective transition from the pro-inflammatory phase to the onset of pro-resolving response^11^. In this context, it is noticeable that some pro-inflammatory molecules involved in the acute phase of inflammation can simultaneously initiate a program for active resolution. For instance, the prototypic pro-inflammatory cytokine IL-1β has been shown to trigger an anti-inflammatory cascade, resulting in the production of IL-10^11^.

Considering differential effects of IL-1β on acute inflammation and its resolution, it can be speculated that the inflammasomes that secrete a mature form of this cytokine may mediate both pro-inflammatory and pro-resolving responses. While activation of the inflammasomes initially mediates pro-inflammatory response, it culminates in the resolution of inflammation and thereby contributes to homeostatic processes^7^. This may account for a dynamic macrophage polarity gradient from pro-inflammatory to anti-inflammatory phenotypes^12^.

The inflammasomes tightly regulate the expression, maturation, and secretion of IL-1β as a master regulator of inflammation. IL-1β, which is mainly generated at the injury or infection sites, induces inflammation-associated gene expression, such as cyclooxygenase-2 and inducible nitric oxide synthase to produce prostaglandin E_2_ and nitric oxide, respectively. Moreover, IL-1β increases the expression of adhesion molecules, such as intercellular adhesion molecule-1 (ICAM-1) in mesenchymal cells and vascular cell adhesion molecule-1 in endothelial cells, which provides favorable condition for the recruitment of inflammatory cells and immunocompetent cells from circulation to damaged or infection sites in cooperation with other chemokines^5^. In addition to IL-1β, another pro-inflammatory cytokine controlled by the inflammasomes is IL-18 which is important for induction of IL-17 expression in Th17 cells, and involved in polarization of T cells toward Th1 or Th2 cells in combination with other cytokines^5^. However, the pathophysiological role of IL-18 in tumor microenvironment has not been well-defined compared to that of IL-1β.

Given its potential roles in the innate immunity, inappropriate regulation of the inflammasomes has a strong association with a variety of inflammatory and autoimmune disorders, including neurodegenerative diseases (e.g., multiple sclerosis, Alzheimer’s disease, and Parkinson’s disease) and metabolic disorders (e.g.,atherosclerosis, type 2 diabetes, and obesity) as well as various human malignancies^9^.

## Involvement of the inflammasomes and their effector molecules in human malignancies

### Breast cancer

Breast cancer is the most commonly diagnosed cancer in women worldwide, and more than 270,000 new cases of breast cancer is estimated to be diagnosed in U.S. women in 2020^13^. Breast cancer is originated from genetic alterations within pre-malignant or malignant cells, and two high-penetrance tumor suppressor genes, *BRCA1* (17q21) and *BRCA2* (13q13), show an autosomal-dominant inheritance pattern^14^. The most frequently mutated and/or amplified genes in the breast tumor cells are *TP53* (41%), *PIK3CA* (30%), *MYC* (20%), *PTEN* (16%), *CCND1* (16%), *ERBB3* (13%), and *FGFR1* (11%)^15^.

Breast cancer cells can invade surrounding tissues and spread to other parts of the body such as bone (30–60%), liver (15–32%), lung (21–32%), and brain (4–10%)^16^, and over 90% of breast cancer-related deaths are associated with metastasis^17^. The breast tumor is characterized by the inflammatory microenvironment, which is supported by the infiltrated immune and inflammatory cells, cytokines, chemokines, and growth factors. Not only proliferation and metastasis of breast cancer cells determine the tumor progressive potential of breast cancer cells, but the complex interactions among many types of cells in the tumor microenvironment can also dictate the fate of breast cancer cells.

**Fig. 2:**
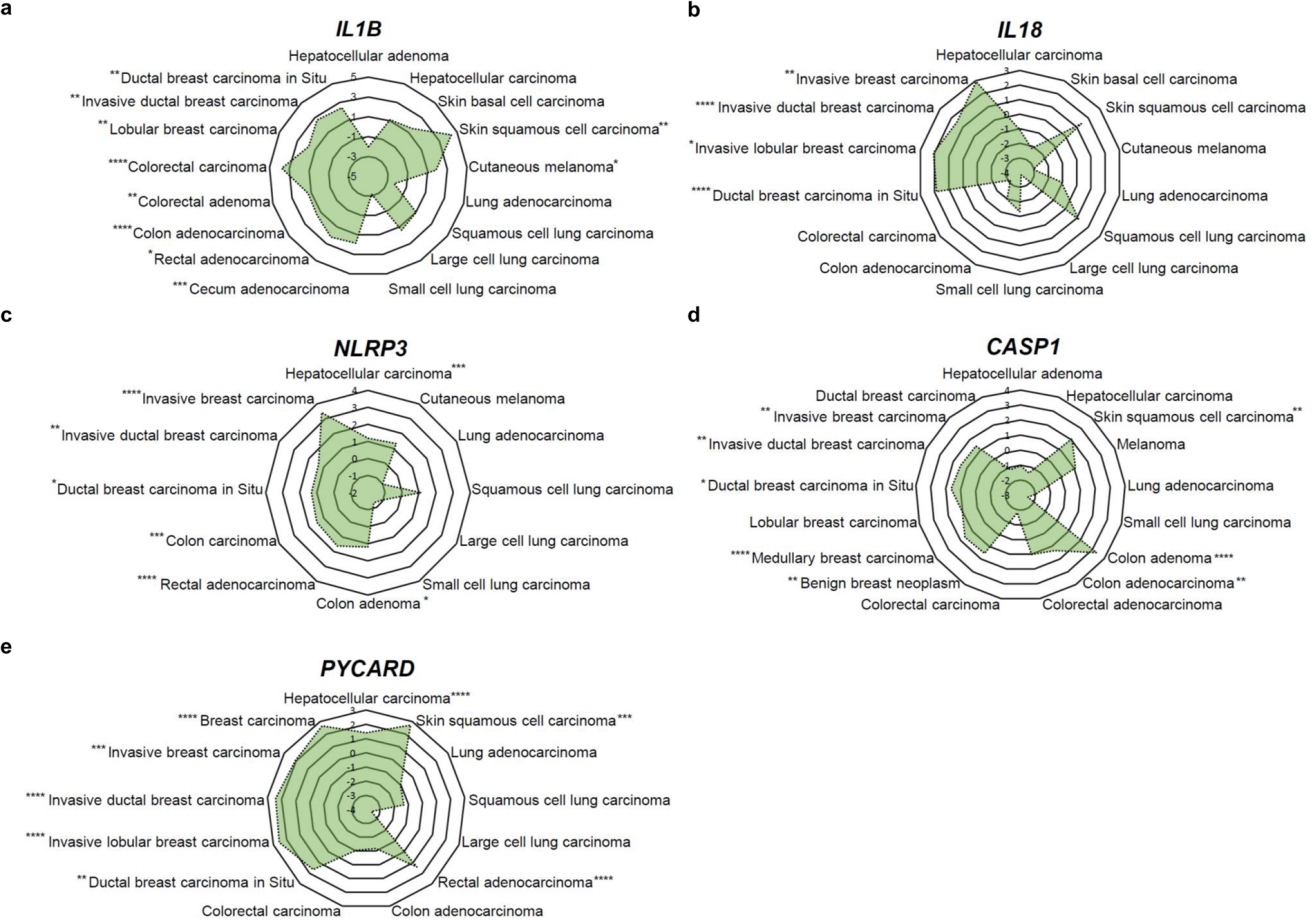
Relative expression of inflammasome components in various cancer. **A** Relative mRNA expression of *IL1B* in various cancers from Oncomine database^104–114^. **B** Relative mRNA expression of *IL18* in various cancers from Oncomine database and The Cancer Genome Atlas (TCGA)^106,108–110,114–120^. **C** Relative mRNA expression of *NLRP3* in various cancers^105,108,109,112,114,116,117,121–123^. **D** Relative mRNA expression of *CASP1* in various cancers^104,106,109,111,112,114,123–130^. **E** Relative mRNA expression of *PYCARD* in various cancers from Oncomine database and TCGA^106,112,115,117,119,122,127,131^. *P < 0.05; **P < 0.01; ***P < 0.001; ****P < 0.0001 (see Supplementary Table S1 for further methodologic details).

The expression of the inflammasome components, such as *IL1B*, *IL18*, *NLRP3*, *PYCARD*, and *CASP1*, is significantly up-regulated in most types of breast cancer compared to that in normal tissues (Fig. 2). In addition, breast cancer patients show highly increased serum levels of IL-1β which is dependent on tumor stage^18,19^. This elevated expression of IL-1β is more likely to be associated with establishment of inflammatory tumor microenvironment and breast cancer progression^18,20–22^. Further, significant correlation was noticed between IL-1β expression and subsequent development of metastasis^23,24^. In a syngeneic orthotopic breast cancer mouse model, IL-1β deficiency failed to induce 4T1 breast cancer cell growth and metastasis, which results from the insufficient recruitment of inflammatory monocytes into tumor site^25^. Consistently, *Nlrp3*^–/–^ and *Casp1*^–/–^ knockout (K/O) mice deficient in inflammasome components showed significantly reduced growth of primary tumor and lung metastasis^22,26^. Disruption of IL-1β–IL-1R signaling by use of a monoclonal IL-1β antibody and an IL-1R antagonist, or by genetic disruption of IL-1R signaling lowered the growth of primary tumor and metastasis to lung and bone in the breast cancer mouse model^20–22,26^.

**Fig. 3:**
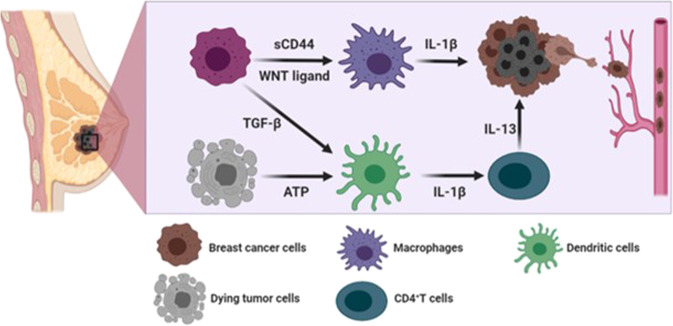
Mechanisms of breast cancer progression mediated through intrinsic inflammasome activation. In breast tumor microenvironment, sCD44 and WNT ligand released by breast cancer cells induce secretion of IL-1β from macrophages. In addition, TGF-β produced by breast cancer cells and ATP released from dying tumor cells stimulate IL-1β secretion from dendritic cells, which promotes IL-13 production in CD4^+^ T cells. This promotes breast cancer progression and metastasis.

Although breast cancer shares the inflammatory characteristics with other types of cancers, this malignancy is rarely associated with inflammation-induced cancer (extrinsic pathway), but rather related to cancer-associated inflammation (intrinsic pathway) for the development of inflammatory microenvironment. Therefore, development of the inflammatory conditions and activation of the inflammasomes largely rely on the interaction among components of the breast tumor microenvironment. Accumulating evidence suggests that antigen-presenting cells, such as dendritic cells and macrophages, are primarily responsible for releasing IL-1β in the breast tumor microenvironment^27–29^. According to Wu et al.^30^, interaction between breast cancer cells and CD11c^+^ dendritic cells induced caspase-1-dependent IL-1β secretion from CD11c^+^ dendritic cells which was stimulated by breast cancer cell membrane-derived transforming growth factor β-1 (Figs. 1 and 3). In addition, ATP released from dying tumor cells upon chemotherapy induced NLRP3 inflammasome activation and IL-1β secretion from dendritic cells through P2X7 purinergic receptor in the breast tumor microenvironment^27^ (Figs. 1 and 3). IL-1β released by dendritic cells promoted differentiation of IL-13-producing CD4^+^ T cells and interferon (IFN)-γ-producing CD8^+^ T cells in breast cancer-bearing humanized mice^27^ (Fig. 3). CD4^+^ T effector cells that express high levels of IL-13 accelerated development of mammary carcinomas and their metastasis to the lung by enhancing pro-tumorigenic potential of tumor-associated macrophages^31,32^ (Fig. 3). IFN-γ derived from CD8^+^ T cells, when primed by IL-1β, reduced the chemotherapeutic efficacy of oxaliplatin^27^. Patients with estrogen receptor (ER)α-positive breast cancer were responsive to tamoxifen, but IL-1β diminished its chemotherapeutic efficacy by down-regulating ERα expression^33^.

In addition to dendritic cells, other types of immune cells also have potential to release IL-1β in the breast tumor microenvironment^27–29^. According to Jang et al.^18^, macrophages are also major cellular sources for IL-1β secretion, and triple-negative breast cancer cells induced ASC oligomerization and speck formation for inflammasome activation in macrophages. Notably, depletion of macrophages decreased serum levels of IL-1β, and alleviated breast cancer progression in a syngeneic orthotopic breast cancer mouse model^18^. The secretion of IL-1β from macrophages is surmised to be mediated by breast cancer cell membrane-derived soluble CD44 (sCD44), as antibody neutralization of sCD44 hampered secretion of IL-1β from macrophages^18^ (Figs. 1 and 3).

WNT ligand-induced IL-1β secretion from macrophages was reported by Wellenstein et al.^34^ (Figs. 1 and 3). Deletion mutation of *TP53* in breast cancer cells conferred secretion of WNT ligands^34^. This, in turn, promoted IL-1β secretion from tumor-associated macrophage, leading to systemic inflammation for breast cancer metastasis^34^. Moreover, bone marrow-derived IL-1β promoted colonization of breast cancer cells in the bone via induction of nuclear factor (NF)-κB/CREB-WNT signaling in breast cancer cells^21^. Obesity-induced NLRC4 inflammasome activation in F4/80^+^/CD11b^+^ macrophages facilitated tumor growth and angiogenesis through upregulation of adipocyte-mediated *Vegfa* expression in the breast tumor microenvironment^35^. Major cellular sources for IL-1β secretion are antigen-presenting cells, but endogenous expression of IL-1β in breast cancer cells also increased metastatic potential, which was correlated with bone metastasis in breast cancer patients^20^.

Although IL-1β released upon activation of inflammasomes is known to induce tumor progression, it also has a tumor suppressive function in the breast tumor microenvironment^36,37^. The metastasis-initiating cancer cells (MICs) play roles in metastasis, recurrence, and therapeutic resistance of cancer cells. According to Castano et al.,^36^ IL-1β can keep MICs in a ZEB1-positive differentiation state, thereby preventing them from generating exceedingly proliferative E-cadherin-positive progeny in a metastatic microenvironment. Thus, colonization and secondary tumor formation are inhibited by sustained IL-1β. In agreement with such differential effects of IL-1β, blockade of its receptor (IL-1R), in combination with paclitaxel, slightly reduced primary breast tumor growth, but potentiated pulmonary metastasis^37^. In another study, *Il-1β*-deficient mice exhibited profound regression of primary tumor growth in a syngeneic orthotopic breast cancer mouse model^25^. Both CD11b^+^ dendritic cells and activated CD8^+^ T cells are mainly considered to exert immunosuppression and anti-tumor immunity by releasing IL-12, IFN-γ, and Granzyme B^25^. *Il-1β*-deficient mice failed to infiltrate inflammatory monocytes, but CD11b^+^ dendritic cells and activated CD8^+^ T cells were more predominant in tumor tissues compared to wild type (WT) mice^25^.

### Colorectal cancer

Colorectal cancer (CRC) is the third most common form of cancer in western countries. CRC is frequently associated with prolonged inflammation in the intestine, as exemplified in patients with inflammatory bowel disease (IBD). Crohn’s disease and ulcerative colitis are two major types of IBD, characterized by a prolonged inflammatory response against luminal bacteria or sustained mucosal danger signals, leading to over-activated immune response in the intestinal tract. Patients with IBD are at high risk for colitis-associated colorectal cancer (CAC). Aspirin and some other non-steroidal anti-inflammatory drugs have been known to reduce risk of CRC, which supports the critical involvement of inflammatory processes in the onset and development of CRC^38^. The CRC microenvironment is composed of epithelial cells, stromal cells, and heterogeneous infiltrating immune and inflammatory cells, including macrophages, dendritic cells, T lymphocytes, B cells, NK cells, regulatory T cells, and myeloid-derived suppressor cells^39^. A dynamic interaction of CRC cells with the surrounding stroma can influence not only development of CRC, but its progression and metastasis.

**Fig. 4:**
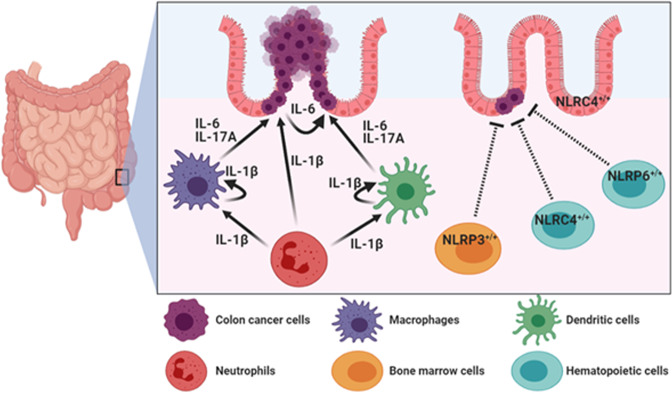
Mechanisms of CRC regulation mediated by extrinsic inflammasome activation. In colorectal tumor microenvironment, IL-1β derived from infiltrated neutrophils, macrophages, and dendritic cells enhances CRC progression through production of IL-6 and IL-17A from lamina propria, macrophages, and dendritic cells. On the other hand, NLRP3 in bone marrow cells, NLRC4 and NLRC6 in hematopoietic cells, and NLRC4 in colon epithelial cells play tumor suppressive roles in CAC development.

Among genes encoding inflammasome components, *IL1B* and *NLRP3* are highly expressed in colon cancer tissues (Fig. 2)^40,41^, and accumulating evidence points to critical roles of the inflammasomes and IL-1β in dextran sulfate sodium (DSS)-induced colitis and azoxymethane (AOM) plus DSS-induced CAC. In this mouse model, AOM is used as an initiator capable of causing DNA damages in intestinal epithelial cells. Repeated administration of DSS in drinking water causes damages to the intestinal epithelial barrier, resulting in the induction of inflammation in the colonic mucosa and promotes development of CRC. In an AOM plus DSS-induced CAC mouse model, mice deficient for the inflammasome components were highly susceptible to development of CAC as demonstrated by increased morbidity, histopathology, and colonic polyp formation^42–45^. In the CAC induced by AOM and DSS, the inflammasomes in hematopoietic-derived cells exerted more potent effects on regulation of intestinal tumorigenesis than those in other types of cells in the intestinal tract, such as epithelial cells, tumor-derived cells, or stromal cells^43,45^. Allen et al.^43^ reported suppressive effects of *Pycard* and *Casp1* as well as *Nlrp3*, on acute and recurring colitis induced by DSS, and found that Pycard and caspase-1 were essential for survival in AOM plus DSS-induced CAC mouse model. Significantly increased colonic lesions and the number of colonic macroscopic polyps were observed in WT mice receiving bone marrow from *Nlrp3* K/O mice when compared with those in mice receiving WT bone marrow^43^. These preclinical data using chimeric animals by adoptive bone marrow transplantation indicate that NLRP3 in hematopoietic-derived cells plays a tumor suppressive role in the CAC microenvironment^43^ (Fig. 4). NLRP6 also showed predominant protective effects in hematopoietic-derived cells against colitis-induced tumorigenesis^45^. *Nlrp6*-deficient hematopoietic-derived cells were more susceptible to inflammation-induced tumorigenesis due to the impairment of resolution of inflammation and repairing damaged epithelium (Fig. 4). NLRP6 in colon epithelial and stromal cells, however, did not show a tumor suppressive effect on the development of CAC, although these cells have higher expression of NLRP6 compared to other types of cells such as lamina propria, granulocytes, and monocyte lineage cells^45^. Similarly, NLRC4 in colon epithelial and hematopoietic cells was found to play protective roles in CAC development^44,46^ (Fig. 4), and the expression of NLRC4 was associated with p53-dependent cancer cell death^47^. Direct induction of *NLRC4* gene expression by p53 enhanced p53-dependent apoptosis^47^. Therefore, NLRC4 in hematopoietic cells and epithelial cells is likely to be important for limiting tumorigenesis^44,46^.

On the other hand, oncogenic potential of IL-1β in the CAC microenvironment has been reported^48,49^. In the CAC pathogenesis, infiltrated neutrophils are considered major cell type responsible for producing IL-1β^48,49^. Therefore, depletion of neutrophils and blockade of IL-1β activity attenuated mucosal damage and reduced the tumor burden^48^ (Fig. 4). IL-1β derived from infiltrated neutrophils induced secretion of IL-6 from lamina propria, resident dendritic cells, and macrophages in the CAC milieu^48^ (Fig. 4). IL-6 has been known to have critical roles in CAC progression, and prolonged disease-free survival was observed in CRC patients with low IL-6 expression in their primary tumors^50^. In addition, IL-1β was required for secretion of IL-17A from intestine-resident myeloid cells in an autocrine or a paracrine manner^49^. Secretion of IL-6 and IL-17A by intestinal myeloid cells (predominantly dendritic cells and macrophages) enhanced proliferation and decreased apoptosis of pre-neoplastic intestinal epithelial cells via the signal transducer and activator of transcription 3 and the NF-κB-dependent signaling pathways in the CAC microenvironment^49,51,52^ (Fig. 4). Overproduction of pro-inflammatory cytokines, such as tumor necrosis factor-α, IL-1β, and IL-17A, is closely associated with the development of CAC^48,49,51–54^.

### Lung cancer

Lung cancer is the leading cause of cancer-related death with a poor 5-year survival of less than 19%^13^. Human lung adenocarcinoma encompasses unique subtypes of lung cancer with distinct cellular and mutational heterogeneity^55^. Importantly, this heterogeneity is not only limited to tumor cells but also spans the tumor microenvironment, including vasculature, extracellular matrix, cancer-associated fibroblast, and infiltrating immune cells. In human non-small-cell-lung cancer (NSCLC), stage-dependent tumor-associated immune cells infiltration indicates strong association of the tumor microenvironment with lung carcinogenesis and prognosis of lung cancer^56,57^.

The relationship between IL-1β and lung cancer has not been well-defined in the context of tumor microenvironment. Indeed, the expression of the inflammasome components in lung cancer tissues did not much differ from those in normal pulmonary tissues (Fig. 2). However, the role for inflammasome components, especially IL-1β attracted attention by the Canakinumab Anti-inflammatory Thrombosis Outcome Study (CANTOS) trial. The CANTOS trial was a randomized, double-blinded, placebo-controlled trial that investigated the use of canakinumab, a monoclonal antibody targeting IL-1β, in high-risk patients with established atherosclerotic disease who had already survived a myocardial infarction. The report showed that among the 129 cases of lung cancer in the follow-up period of 3.7 years (median), the incidence of lung cancer was significantly less frequent in the groups receiving the 150 and 300 mg of canakinumab, not in the group receiving 50 mg. The CANTOS trial suggests the role for IL-1β in lung cancer progression. Based on the CANTOS trial, three parts of phase III CANOPY clinical trials to evaluate canakinumab in patients with mid- to late-stage of NSCLC have been undertaken.

In lung cancer, tumor-associated macrophages have been suggested as the principal cellular sources of IL-1β secretion, and macrophage depletion alleviated *N*-methyl-*N*-nitroso-urea-induced pulmonary cancer development^58^. In addition, tumor-associated macrophages isolated from lung tumor bearing mice exhibited increased production of IL-1β when stimulated with LPS plus ATP^58^. Similarly, peripheral blood leukocytes from patients with primary lung cancer released more IL-1β and IL-18 than did those from healthy individuals^59^. In addition, an important role of IL-1β derived from neutrophils was elucidated in relation to the impaired efficacy of NF-κB inhibitor against lung carcinogenesis^60^. Thus, an NF-κB inhibitor shows limited effects for lung cancer treatment, although NK-κB signaling in epithelial cells has been known to play an essential role in lung carcinogenesis^60^. Specific inhibition of NF-κB in myeloid cells increased IL-1β processing in neutrophils, thereby stimulating proliferative activity of epithelial cells^60^. In lung cancer patients who received the NF-κB inhibitor, the serum levels of IL-1β were increased, and co-treatment of an IL-1R antagonist and the NF-κB inhibitor decreased formation of lung cancer in mice^60^.

According to Carmi et al.^61^, secretion of IL-1β mainly released from myeloid cells in the lung tumor microenvironment is prerequisite for the recruitment and activation of IL-17 producing γ/δ T cells. They also suggested the importance of IL-1β balance to prevent stimulation of cancer progression in the lung tumor microenvironment^61^. Specifically, poor prognosis of lung cancer and reduced T cell activity have been observed in both IL-1β K/O and IL-1R antagonist K/O mice^61^. The absence of IL-1 signaling in IL-1β K/O mice promoted regulatory T cell development that suppressed anti-tumor immunity^61^. On the other hand, the increased activity of IL-1 by using an IL-1R antagonist K/O mice enhanced accumulation of myeloid-derived suppressor cells, resulting in suppression of T cell function in the lung tumor microenvironment^61^. Except hematopoietic cells, IL-1β overexpressing lung cancer cells acquired an aggressive phenotype for cancer development by increasing the expression of ICAM-1 and matrix metalloproteinase-2^62^. Moreover, IL-1β-overexpressing cells, when injected intravenously, were more likely to metastasize to the lung, which was blocked by anti-IL-1β antibody treatment^62^.

### Skin cancer

The most important environmental risk factor for skin cancer is ultraviolet (UV) radiation, which has been known to cause DNA damage, immunosuppression, and inflammation^63,64^. Basal cell carcinoma, the most common type of skin cancers, accounts for up to 80% of all malignant skin cancers and higher levels of pro-inflammatory cytokines (e.g., IL-1β, IL-5, and IL-6), compared to those in squamous cell carcinomaoma^65^. The most aggressive form of skin cancer is melanoma which is characterized by upregulation of several pro-inflammatory cytokines, including IL-6, IL-8, CCL5, and IL-1β, the expression of which can be regulated by IL-1β^5,64,66^. In addition, expression of the inflammasome components, such as *IL1B*, *PYCARD*, and *CASP1*, was detected at higher levels in squamous cell carcinoma than in normal skin tissues (Fig. 2).

In skin cancer, UV radiation is a powerful inducer of IL-1β, and melanoma-derived IL-1β is more likely to promote tumor growth, angiogenesis, invasion, and metastasis^67–73^ (Fig. 1). However, the roles of the inflammasomes and IL-1β in skin cancer vary, depending on the types of cells. Several studies using a two-stage mouse skin carcinogenesis model revealed that the inflammasomes and IL-1 have dual functions in the development of inflammation-induced skin carcinogenesis. According to the study by Drexler et al., *Il-1r1*^−/−^ and *Casp1*^−/−^ mice treated with 7,12-dimethylbenz(*a*)anthracene (DMBA) as an initiator and 12-*O*-tetradecanoylphorbol-13-acetate (TPA) as a promoter showed the reduced incidence and the multiplicity of skin tumors compared to WT mice, suggesting the tumor supportive role of the IL-1β–IL-1R axis in two-stage skin carcinogenesis^74^. On the other hand, *Asc*^−/−^ mice showed no significant functional difference in the tumor burden and the incidence compared to WT mice in response to DMBA and TPA treatment^74^.

Notably, ASC showed completely different behavior in either keratinocytes or myeloid cells in tissue-specific conditional K/O mice^74^. Mice with exclusive deletion of ASC in myeloid cells developed fewer tumors than WT mice, but the number and the incidence of tumors were higher in mice deficient for ASC in keratinocytes, although the onset of tumor formation was delayed for a few weeks when compared to WT control^74^. The results from ASC tissue-specific conditional K/O mice imply cell-type-dependent dual functions of the inflammasome components in a skin carcinogenesis mouse model. Consistent with these pleiotropic results, ASC expressed by primary melanoma exerted an inhibitory effect on tumorigenesis by suppressing IκB kinase α/β phosphorylation and NF-κB transcriptional activity^75^. On the other hand, relatively up-regulated expression of ASC in metastatic melanoma increased NF-κB activity and the inflammasome-mediated IL-1β secretion^75^. Knock-down of *NLRP1* in melanoma cells attenuated their tumor-promoting properties through regulation of the inflammasomes and the apoptotic pathway in both in vitro and in vivo^76^. Considering the absence of NLRP3 expression in keratinocytes, myeloid cells appear to have a major responsibility for the development of NLRP3 inflammasome-mediated skin cancer development and progression^29,77^.

### Liver cancer

Hepatocellular carcinoma (HCC) is the most common primary malignant tumor of the liver. Hepatitis C virus (HCV) infection is a main cause of advanced hepatic fibrosis and cirrhosis, associated with significantly increased risk of HCC development. Kupffer cells (resident macrophages in liver) and HCC cells have been known as major sources of IL-1β secretion in HCV-mediated progression of HCC^78,79^. HCV induces assembly of the NLRP3 inflammasome complex in human HCC cells, and up-regulated expression of *NLRP3* and *PYCARD* was observed in HCC tissues (Fig. 2). HCV-induced NLRP3 inflammasome activation in Kupffer cells led to increased serum levels of IL-1β in HCV-infected patients^78,79^. Necrotic debris of HCC cells formed under hypoxic conditions also induced secretion of IL-1β from macrophages in the HCC microenvironment^80^. Secretion of IL-1β enhanced epithelial-mesenchymal transition (EMT) and metastasis of HCC cells through stabilization of hypoxia-inducible factor-1α^80^. On the contrary, the up-regulated expression of IL-1β and NF-κB extended disease-free survival of HCC patients after hepatectomy^81^. Likewise, the expression of NLRP3 inflammasome components, including NLRP3, ASC, caspase-1, and IL-1β, showed inverse correlation with pathological grades and clinical stages in the HCC patient tissues. Compared with peritumoral non-cancerous liver tissues, NLRP3 inflammasome components were down-regulated in hepatic parenchymal cells in human HCC, which was associated with more advanced clinical stages^82^. During the development of HCC, massive loss of hepatocytes is accompanied by chronic hepatic inflammation. Dying hepatocytes release danger signals, and their accumulation causes liver damage, which may further increase the risk of liver cancer^83^. NLRP3 inflammasomes have essential roles in sensing danger signals. Therefore, loss of NLRP3 inflammasome components during the development of HCC leads to failure of sensing danger signals, which may result in manifestation of an aggressive phenotype of liver cancer development.

## Therapeutic approaches and clinical trials

For the therapeutic purpose, attempt has been made to repress the aberrant activation of the inflammasomes. Although some natural compounds have been shown to inhibit overactivation of the inflammasomes or their components, most studies aimed to test their ability to ameliorate simply inflammatory symptoms, not to prevent/treat cancer (Table 2)^84,85^. Therefore, it will be worthwhile determining chemopreventive or chemotherapeutic effects of natural and synthetic compounds exerted through regulation of the inflammasomes. However, the tumor microenvironment comprises various types of cells that interact one another. Therefore, the dynamic interaction among various cells in the tumor microenvironment should be considered for the inflammasomes-targeted cancer prevention/treatment.

**Table 2:** Regulation of inflammasomes by natural compounds.

Natural compounds	Mechanisms	Ref.
Curcumin	Reduced expression of NLRP3, inhibition of caspase-1 cleavage, and secretion of IL-1β	^132^
Curcumin	Inhibition IL-1β secretion in bone marrow-derived macrophages (BMDMs) by preventing K^+^ efflux, ROS generation, and cathepsin B release	^133,134^
Resveratrol	Inhibition of NLRP3 inflammasome via preventing assembly of ASC and NLRP3 on the mitochondria and ER	^135^
*cis*-resveratrol	Inhibition of IL-1β secretion by suppression of P2X7R and ROS production	^136^
Sulforaphane	Inhibition of Aβ1-42-induced caspase-1 dependent inflammasome activation via inhibition of STAT-1 phosphorylation and activation of the Nrf2/HO-1 signaling cascade	^137^
Sulforaphane	Reduced monosodium urate (MSU) crystal-induced IL-1β secretion in peritonitis model, and inhibited NLRP1b, NLRP3, NAIP5/NLRC4 and AIM2 inflammasomes in BMDMs	^138^

In addition to natural and synthetic compounds, some biological agents (e.g., anakinra and canakinumab) targeting IL-1β or its receptor are now used to treat several inflammatory or autoimmune diseases, such as rheumatoid arthritis. IL-1β mainly contributes to the establishment of systemic inflammation in the tumor microenvironment and tumor progression^86^. Based on these findings, several trials have been launched using IL-1β neutralizing antibody and IL-1R antagonists (Table 3). Antibody neutralization of IL-1β and use of IL-1R antagonists showed significant anti-cancer effects in preclinical studies, and their safety was confirmed. According to Holen et al., an IL-1R1 antagonist (anakinra) significantly reduced the growth of primary tumor and its bone metastasis in a breast cancer mouse model^26^. Although anakinra did not induce apoptosis of tumor cells, it significantly inhibited cancer cell proliferation and angiogenesis^26^. In agreement with these results, Wu et al.^30^ reported inhibitory effects of an IL-1R1 antagonist on breast cancer progression. Notably, anakinra treatment prevented breast cancer progression with a substantial decrease in the proportion of IL-13 producing tumor-infiltrating CD4^+^ T cells and a concomitant increase of IFN-γ producing CD4^+^ T cells in a humanized mouse model^30^. In other studies, administration of anakinra suppressed growth and lymph node metastasis of the LNM35 human lung tumor xenograft in mice^87^. In addition, a recombinant human IL-1R antagonist plus one of the standard chemotherapy regimens, gemcitabine, significantly reduced tumor burden when compared to the gemcitabine treatment alone in a pancreatic ductal adenocarcinoma orthotopic xenograft mouse model^88^.

**Table 3:** Pharmaceutical target of inflammasome in cancer.

Therapeutic agents (Trade name)	Targets	Mechanisms of action	Cancer type	Status	Ref.
Anakinra (Kineret)	IL-1 receptor	IL-1R antagonist	Metastatic breast cancer	Phase I	NCT01802970
Anakinra (Kineret)	IL-1 receptor	IL-1R antagonist	Metastatic CRC	Phase II	NCT02090101
Anakinra (Kineret)	IL-1 receptor	IL-1R antagonist	Multiple myeloma & plasma cell neoplasm	Phase II	NCT00635154
Canakinumab (Ilaris)	IL-1β	Anti-IL-1β monoclonal antibody	CRC, triple-negative breast cancer, NSCLC	Phase I	NCT02900664
Gevokizumab	IL-1β	Anti-IL-1β monoclonal antibody	Metastatic CRC, gastroesophageal cancer, renal cell carcinoma	Phase I	NCT03798626
MCC950	NLRP3 inflammasome	Inhibition of ASC oligomerization	Head and neck squamous cell carcinoma	Preclinical	^139^
Oxidized ATP	P2X7 receptor	Selective P2X7 antagonist	P2X7-expressing tumors, melanoma	NA	^139,140^

In the Palucka pilot clinical trial with 11 HER2-negative metastatic breast cancer patients, 100 mg/daily of anakinra was subcutaneously administered for a 2-week run-in treatment period^30^. This was followed by continuous daily anakinra administration along with one of the standard chemotherapy regimens for HER2-negative breast cancer patients for a median duration of 4 months. In this preclinical study, 2 of 11 patients had a considerably reduced tumor size, 4 had stable disease, 2 stopped anakinra administration due to injection site reactions, and the other 3 had progressive disease. Some patients showed reduced pain and increased quality of life on anakinra plus chemotherapy, and anakinra also reduced chemotherapy-associated “sickness syndrome”. Canakinumab was approved for use as an anti-IL-1β neutralizing mAb by US FDA in 2009. In a randomized, double-blind, placebo-controlled trial with 10,061 atherosclerosis patients, canakinumab showed a significantly reduced incidence and a mortality of lung cancer compared to the placebo group^89^.

In a phase 2 clinical study, the activity and safety of 5-fluorouracil plus bevacizumab and anakinra were tested in unresectable metastatic CRC patients^90^. The patients were treated with a simplified folinic acid plus 5-fluorouracil regimen and bevacizumab for every 2 weeks, and anakinra was injected subcutaneously once daily for 2 months. In this study, the median progression-free survival (PFS) and the overall survival (OS) were 5.4 (95% CI, 3.6–6.6) and 14.5 months (95% CI, 9–20.6), respectively, and no treatment-related deaths or serious adverse effects were observed^90^. Although it is hard to define the direct effects of this combination therapy in metastatic CRC patients due to the absence of a comparable group, the PFS and OS were extended compared to the Regorafenib study [PFS: 2.8 months (95% CI 1.4–3.7) versus 1.8 months (95% CI 1.3–1.7), median OS: 6.4 months (95% CI 3.6–11.8) versus 5.0 months (95% CI 2.8–10.4)] and TAS-102 trial [median PFS: 2.0 months (95% CI, 1.9 to 2.1) in the TAS-102 group and 1.7 months (95% CI, 1.7 to 1.8) in the placebo group, median OS improved from 5.3 months (95% CI, 4.6–6) to 7.1 months (95% CI, 6.5–7.8)]. Given the extended PFS and OS, this combination therapy can be suggested as an option for refractory metastatic CRC patients^90–92^. Based on these findings, further studies will be necessary to achieve better clinical results qualified for use in cancer patients. In addition, the timing, duration, and dosing for the inflammasome inhibition should be considered as important factors, since excessive suppression of the inflammasomes can cause severe immunosuppression, which may increase the risk of serious infections.

There are several trials that utilize inflammasome components as a biomarker in chronic diseases and systemic injuries, including stroke^93^, traumatic brain injury^94^, multiple sclerosis^95^, and Alzheimer’s disease^96^. Although *IL1B* gene polymorphism is associated with the risk of lung cancer^97^, inflammasome components are barely used for cancer diagnosis due to its non-specificity. As previously discussed, inflammasomes are not only expressed in cancer, but also activated in many types of inflammation or immune-associated disorders. Nonetheless, the inflammasome components can be considered as prognostic markers of cancer, but the changes in their activation and overexpression of their components according to the status of cancer progression need systematic validation.

## Conclusion and perspectives

The inflammasomes are involved in the whole range of tumorigenesis. The consequence of aberrantly activated inflammasomes in cancer development and progression varies depending on types of tumors, location of inflammasomes, cells, stages of cancer, and profile and composition of the tumor microenvironment (Table 1). The roles of inflammasomes, especially those of hematopoietic lineage, in stimulating tumor progression have been well demonstrated in CRC and breast cancer^27,42,45^. In CRC development, NLRP3 and NLRC4 in hematopoietic-derived cells have differential effects on tumor formation^43,46^. Moreover, NLRP6 showed differential roles depending on cell types for its expression in CAC^45^. Ironically, NLRP3 and NLRP6 expressed in bone marrow cells play tumor suppressive roles^43,45^, but their byproduct, IL-1β is more likely to induce tumor progression in the CRC microenvironment^48^. Such discrepancy may imply the importance of the inflammasomes and IL-1β and their balance in the tumor microenvironment. In inflammation-induced cancer, deficient or impaired activation of the inflammasomes and expression/processing of their components may provoke failure in engaging inflammation and its resolution in an early stage of chemically induced carcinogenesis^43^. Failure of systemic inflammatory responses, such as inflammation initiation, amplification, and resolution, leads to severe tissue damages, accumulation of PAMPs or DAPMs, and delayed tissue repair, resulting in tumor development^83^. The roles and effects of the inflammasomes can be influenced by various factors, and more detailed study for its expression pattern and interaction with microenvironment is needed.

Pyroptosis is a highly inflammatory form of cell death characterized by pore-formation, cell swelling, plasma membrane rupture, and intracellular contents release in immune cells as well as some epithelial cells^8^. This unique process is initiated by inflammasome activation upon recognition of PAMPs or DAMPs^8^. The association between pyroptosis and cancer is complex and not clearly defined. Thus, the effects of pyroptosis on cancer development and progression vary, based on the types of tissues and genetic backgrounds^98–100^. On the one hand, pyroptosis inhibits proliferation and migration of cancer cells^101,102^. Thus, expression of some pyroptotic inflammasomes has been found to decreased in cancer cells. Recent studies have focused on simvastatin and non-coding RNA molecules inducing pyroptotic cell death in several types of cancers^101,102^. On the other hand, pyroptosis can provide a suitable microenvironment for growth of cancer cells and thereby stimulates tumor growth by releasing IL-1β and IL-18.

In this review, we highlighted the differential roles of inflammasomes and its effector molecules, particularly IL-1β, in tumor development and progression. Inflammasomes and inflammation components have both tumor suppressive and promoting functions^103^. These depend on cell types, malignancy types, downstream effector molecules, and stages of cancers. More systematic and integrative studies on the roles for inflammasomes as well as IL-1β and IL-18 in specific stages of cancer, from tumor initiation to progression and metastasis, merit further investigations.

### Reporting summary

Further information on research design is available in the Nature Research Reporting Summary linked to this article.

## Supplementary information

Supplementary Table S1

Reporting Summary

## Data Availability

The data used to create Fig. 2 were obtained from Oncomine database using the free, publicly available databases or a free, publicly available web-based analytical platform, respectively (see Supplementary Table S1).
