# Supplementary material for: Dynamic roles of inflammasomes in inflammatory tumor microenvironment
Source: NPJ Precis Oncol. 2021 Mar 8;5:18. doi: 10.1038/s41698-021-00154-7 (PMC7940484; doi:10.1038/s41698-021-00154-7)
Supplement: Supplementary file 1 — Supplementary Table S1 [file 41698_2021_154_MOESM1_ESM.docx]

**Supplementary information**

**Supplementary Table S1. List of reference sources from Oncomine database platform.**

| **Inflammasome components** | **Types of cancer** | **Sources (DOI)** |
| --- | --- | --- |
| IL1B | Hepatocellular Adenoma (n=3) | 10.1091/mbc.02-02-0023 |
| IL1B | Hepatocellular Carcinoma (n=38) | 10.2119/molmed.2008.00110 |
| IL1B | Skin Basal Cell Carcinoma (n=15) | 10.1186/1755-8794-1-13 |
| IL1B | Skin Squamous Cell Carcinoma (n=5) | 10.1186/1476-4598-5-30 |
| IL1B | Cutaneous Melanoma (n=45) | 10.1158/1078-0432.CCR-05-0683 |
| IL1B | Lung Adenocarcinoma (n=132) | 10.1073/pnas.191502998 |
| IL1B | Squamous Cell Lung Carcinoma (n=21) | 10.1073/pnas.191502998 |
| IL1B | Large Cell Lung Carcinoma (n=4) | 10.1073/pnas.191502998 |
| IL1B | Small Cell Lung Carcinoma (n=6) | 10.1073/pnas.191502998 |
| IL1B | Cecum Adenocarcinoma (n=17) | 10.1186/gb-2007-8-7-r131 |
| IL1B | Rectal Adenocarcinoma (n=8) | 10.1186/gb-2007-8-7-r131 |
| IL1B | Colon Adenocarcinoma (n=41) | 10.1186/gb-2007-8-7-r131 |
| IL1B | Colorectal Adenoma (n=56) | 10.2353/ajpath.2008.070851 |
| IL1B | Colorectal Carcinoma (n=36) | 10.1371/journal.pone.0013091 |
| IL1B | Lobular Breast Carcinoma (n=14) | 10.1091/mbc.e03-11-0786 |
| IL1B | Invasive Ductal Breast Carcinoma (n=9) | 10.1186/bcr2222 |
| IL1B | Ductal Breast Carcinoma *In Situ* (n=11) | 10.1186/bcr2222 |
| IL18 | Hepatocellular Carcinoma (n=225) | [10.1158/0008-5472.CAN-10-2607](https://doi.org/10.1158/0008-5472.CAN-10-2607) |
| IL18 | Skin Basal Cell Carcinoma (n=15) | [10.1186/1755-8794-1-13](https://doi.org/10.1186/1755-8794-1-13) |
| IL18 | Skin Squamous Cell Carcinoma (n=11) | [10.1186/1755-8794-1-13](https://doi.org/10.1186/1755-8794-1-13) |
| IL18 | Cutaneous Melanoma (n=45) | [10.1158/1078-0432.CCR-05-0683](https://doi.org/10.1158/1078-0432.CCR-05-0683) |
| IL18 | Lung Adenocarcinoma (n=58) | [10.1371/journal.pone.0001651](https://doi.org/10.1371/journal.pone.0001651) |
| IL18 | Squamous Cell Lung Carcinoma (n=21) | [10.1073/pnas.191502998](https://doi.org/10.1073/pnas.191502998) |
| IL18 | Large Cell Lung Carcinoma (19) | [10.1371/journal.pone.0010312](https://doi.org/10.1371/journal.pone.0010312) |
| IL18 | Small Cell Lung Carcinoma (6) | [10.1073/pnas.191502998](https://doi.org/10.1073/pnas.191502998) |
| IL18 | Colon Adenocarcinoma (n=41) | [10.1186/gb-2007-8-7-r131](https://doi.org/10.1186/gb-2007-8-7-r131) |
| IL18 | Colorectal Carcinoma (n=70) | [10.1007/s10585-010-9305-4](https://doi.org/10.1007/s10585-010-9305-4) |
| IL18 | Ductal Breast Carcinoma in Situ (n=11) | [10.1186/bcr2222](https://doi.org/10.1186/bcr2222) |
| IL18 | Invasive Lobular Breast Carcinoma (n=5) | [10.1073/pnas.0500904102](https://doi.org/10.1073/pnas.0500904102) |
| IL18 | Invasive Ductal Breast Carcinoma (n=389) | TCGA |
| IL18 | Invasive Breast Carcinoma (n=154) | [10.1007/s10549-011-1412-7](https://doi.org/10.1007/s10549-011-1412-7) |
| NLRP3 | Hepatocellular Carcinoma (n=38) | [10.2119/molmed.2008.00110](https://doi.org/10.2119/molmed.2008.00110) |
| NLRP3 | Cutaneous Melanoma (n=45) | [10.1158/1078-0432.CCR-05-0683](https://doi.org/10.1158/1078-0432.CCR-05-0683) |
| NLRP3 | Lung Adenocarcinoma (n=58) | [10.1371/journal.pone.0001651](https://doi.org/10.1371/journal.pone.0001651) |
| NLRP3 | Squamous Cell Lung Carcinoma (n=5) | [10.1093/bioinformatics/bti688](https://doi.org/10.1093/bioinformatics/bti688) |
| NLRP3 | Large Cell Lung Carcinoma (n=19) | [10.1371/journal.pone.0010312](https://doi.org/10.1371/journal.pone.0010312) |
| NLRP3 | Small Cell Lung Carcinoma (n=6) | [10.1073/pnas.191502998](https://doi.org/10.1073/pnas.191502998) |
| NLRP3 | Colon Adenoma (n=5) | [10.1371/journal.pone.0013091](https://doi.org/10.1371/journal.pone.0013091) |
| NLRP3 | Rectal Adenocarcinoma (n=65) | [10.1002/gcc.20811](https://doi.org/10.1002/gcc.20811) |
| NLRP3 | Colon Carcinoma (n=5) | [10.1371/journal.pone.0013091](https://doi.org/10.1371/journal.pone.0013091) |
| NLRP3 | Ductal Breast Carcinoma in Situ (n=11) | [10.1186/bcr2222](https://doi.org/10.1186/bcr2222) |
| NLRP3 | Invasive Ductal Breast Carcinoma (n=9) | [10.1186/bcr2222](https://doi.org/10.1186/bcr2222) |
| NLRP3 | Invasive Breast Carcinoma (n=53) | [10.1038/nm1764](https://doi.org/10.1038/nm1764) |
| CASP1 | Hepatocellular Adenoma (n=3) | [10.1091/mbc.02-02-0023](https://doi.org/10.1091/mbc.02-02-0023) |
| CASP1 | Hepatocellular Carcinoma (n=104) | [10.1091/mbc.02-02-0023](https://doi.org/10.1091/mbc.02-02-0023) |
| CASP1 | Skin Squamous Cell Carcinoma (n=11) | [10.1186/1755-8794-1-13](https://doi.org/10.1186/1755-8794-1-13) |
| CASP1 | Melanoma (n=6) | [10.1073/pnas.0501564102](https://doi.org/10.1073/pnas.0501564102) |
| CASP1 | Lung Adenocarcinoma (n=132) | [10.1073/pnas.191502998](https://doi.org/10.1073/pnas.191502998) |
| CASP1 | Small Cell Lung Carcinoma (n=6) | [10.1073/pnas.191502998](https://doi.org/10.1073/pnas.191502998) |
| CASP1 | Colon Adenoma (n=5) | [10.1371/journal.pone.0013091](https://doi.org/10.1371/journal.pone.0013091) |
| CASP1 | Colon Adenocarcinoma (n=50) | 10.1002/ijc.22975 |
| CASP1 | Colorectal Adenocarcinoma (n=56) | [10.2353/ajpath.2008.070851](https://doi.org/10.2353/ajpath.2008.070851) |
| CASP1 | Colorectal Carcinoma (n=70) | [10.1158/1078-0432.CCR-06-1633](https://doi.org/10.1158/1078-0432.CCR-06-1633) |
| CASP1 | Benign Breast Neoplasm (n=3) | [10.1038/nature10983](https://doi.org/10.1038/nature10983) |
| CASP1 | Medullary Breast Carcinoma (n=32) | [10.1038/nm1764](https://doi.org/10.1038/nm1764) |
| CASP1 | Lobular Breast Carcinoma (n=4) | [10.1038/35021093](https://doi.org/10.1038/35021093) |
| CASP1 | Ductal Breast Carcinoma in Situ (n=11) | [10.1186/bcr2222](https://doi.org/10.1186/bcr2222) |
| CASP1 | Invasive Ductal Breast Carcinoma (n=7) | [10.1038/nature06188](https://doi.org/10.1038/nature06188) |
| CASP1 | Invasive Breast Carcinoma (n=53) | [10.1038/nm1764](https://doi.org/10.1038/nm1764) |
| CASP1 | Ductal Breast Carcinoma (n=88) | [10.1073/pnas.191367098](https://doi.org/10.1073/pnas.191367098) |
| PYCARD | Hepatocellular Carcinoma (n=225) | [10.1158/0008-5472.CAN-10-2607](https://doi.org/10.1158/0008-5472.CAN-10-2607) |
| PYCARD | Skin Squamous Cell Carcinoma (n=11) | [10.1186/1755-8794-1-13](https://doi.org/10.1186/1755-8794-1-13) |
| PYCARD | Lung Adenocarcinoma (n=226) | [10.1158/0008-5472.CAN-11-1403](https://doi.org/10.1158/0008-5472.CAN-11-1403) |
| PYCARD | Squamous Cell Lung Carcinoma (n=27) | [10.1371/journal.pone.0010312](https://doi.org/10.1371/journal.pone.0010312) |
| PYCARD | Large Cell Lung Carcinoma (n=19) | [10.1371/journal.pone.0010312](https://doi.org/10.1371/journal.pone.0010312) |
| PYCARD | Rectal Adenocarcinoma (n=65) | [10.1002/gcc.20811](https://doi.org/10.1002/gcc.20811) |
| PYCARD | Colon Adenocarcinoma (n=101) | TCGA |
| PYCARD | Colorectal Carcinoma (n=36) | [10.1371/journal.pone.0013091](https://doi.org/10.1371/journal.pone.0013091) |
| PYCARD | Ductal Beast Carcinoma in situ (n=3) | [10.1073/pnas.0500904102](https://doi.org/10.1073/pnas.0500904102) |
| PYCARD | Invasive Lubular Breast Carcinoma (n=148) | [10.1038/nature10983](https://doi.org/10.1038/nature10983) |
| PYCARD | Invasive Ductal Breast Carcinoma (n=1556) | [10.1038/nature10983](https://doi.org/10.1038/nature10983) |
| PYCARD | Invasive Breast Carcinoma (n=21) | [10.1038/nature10983](https://doi.org/10.1038/nature10983) |
| PYCARD | Breast Carcinoma (n=14) | [10.1038/nature10983](https://doi.org/10.1038/nature10983) |

**Method for Oncomine database processing:**

The expression levels of *IL1B*, *IL18*, *NLRP3*, *CASP1*, and *PYCARD* mRNA were analyzed in various types of cancers and matched normal tissues based on the Oncomine Platform ([www.oncomine.org](http://www.oncomine.org)) and TCGA. The gene expression in various types of cancers was normalized by that in matched normal tissues. The following filters were used: Genes, *IL1B*, *IL18*, *NLRP3*, *CASP1*, and *PYCARD*; differential analysis, cancer vs. normal analysis. All statistical methods and statistical values were obtained directly from the corresponding database.
